# Statement of Retraction: Comprehensive analysis of PPPCs family reveals the clinical significance of PPP1CA and PPP4C in breast cancer

**DOI:** 10.1080/21655979.2026.2745471

**Published:** 2026-11-05

We, the Editors and Publisher of the journal *Bioengineered*, have retracted the following article from publication.

Xie, W., Sun, Y., Zeng, Y., Hu, L., Zhi, J., Ling, H., … Gao, M. (2022). Comprehensive analysis of PPPCs family reveals the clinical significance of PPP1CA and PPP4C in breast cancer. *Bioengineered*, 13(1), 190–205. https://doi.org/10.1080/21655979.2021.2012316

Since publication, significant concerns have been raised by a third party about the integrity of the data and reported results in the article.

When approached for an explanation, the authors have not been able to provide their original data or any necessary supporting information and so these serious concerns remain unaddressed.

As verifying the validity of published work is core to the integrity of the scholarly record, we are therefore retracting the article. The authors have been informed of this decision. The author(s) agree with the retraction.

We have been informed in our decision-making by our policy on publishing ethics and integrity and COPE guidelines.

The retracted article will remain online to maintain the scholarly record and will be digitally watermarked on each page as ‘Retracted’.

